# Impact of Rutin and Other Phenolic Substances on the Digestibility of Buckwheat Grain Metabolites

**DOI:** 10.3390/ijms23073923

**Published:** 2022-04-01

**Authors:** Ivan Kreft, Mateja Germ, Aleksandra Golob, Blanka Vombergar, Francesco Bonafaccia, Zlata Luthar

**Affiliations:** 1 Nutrition Institute, Tržaška 40, SI-1000 Ljubljana, Slovenia; 2 Biotechnical Faculty, University of Ljubljana, SI-1000 Ljubljana, Slovenia; mateja.germ@bf.uni-lj.si (M.G.); aleksandra.golob@bf.uni-lj.si (A.G.); francesco.bonafaccia29@gmail.com (F.B.); 3 The Education Centre Piramida Maribor, SI-2000 Maribor, Slovenia; blanka.vombergar@guest.arnes.si

**Keywords:** buckwheat, rutin, quercetin, protein, starch, nutrition, flavonoid

## Abstract

Tartary buckwheat (*Fagopyrum tataricum* Gaertn.) is grown in eastern and central Asia (the Himalayan regions of China, Nepal, Bhutan and India) and in central and eastern Europe (Luxemburg, Germany, Slovenia and Bosnia and Herzegovina). It is known for its high concentration of rutin and other phenolic metabolites. Besides the grain, the other aboveground parts of Tartary buckwheat contain rutin as well. After the mixing of the milled buckwheat products with water, the flavonoid quercetin is obtained in the flour–water mixture, a result of rutin degradation by rutinosidase. Heating by hot water or steam inactivates the rutin-degrading enzymes in buckwheat flour and dough. The low buckwheat protein digestibility is due to the high content of phenolic substances. Phenolic compounds have low absorption after food intake, so, after ingestion, they remain for some time in the gastrointestinal tract. They can act in an inhibitory manner on enzymes, degrading proteins and other food constituents. In common and Tartary buckwheat, the rutin and quercetin complexation with protein and starch molecules has an impact on the in vitro digestibility and the appearance of resistant starch and slowly digestible proteins. Slowly digestible starch and proteins are important for the functional and health-promoting properties of buckwheat products.

## 1. Introduction

**Figure 1:**
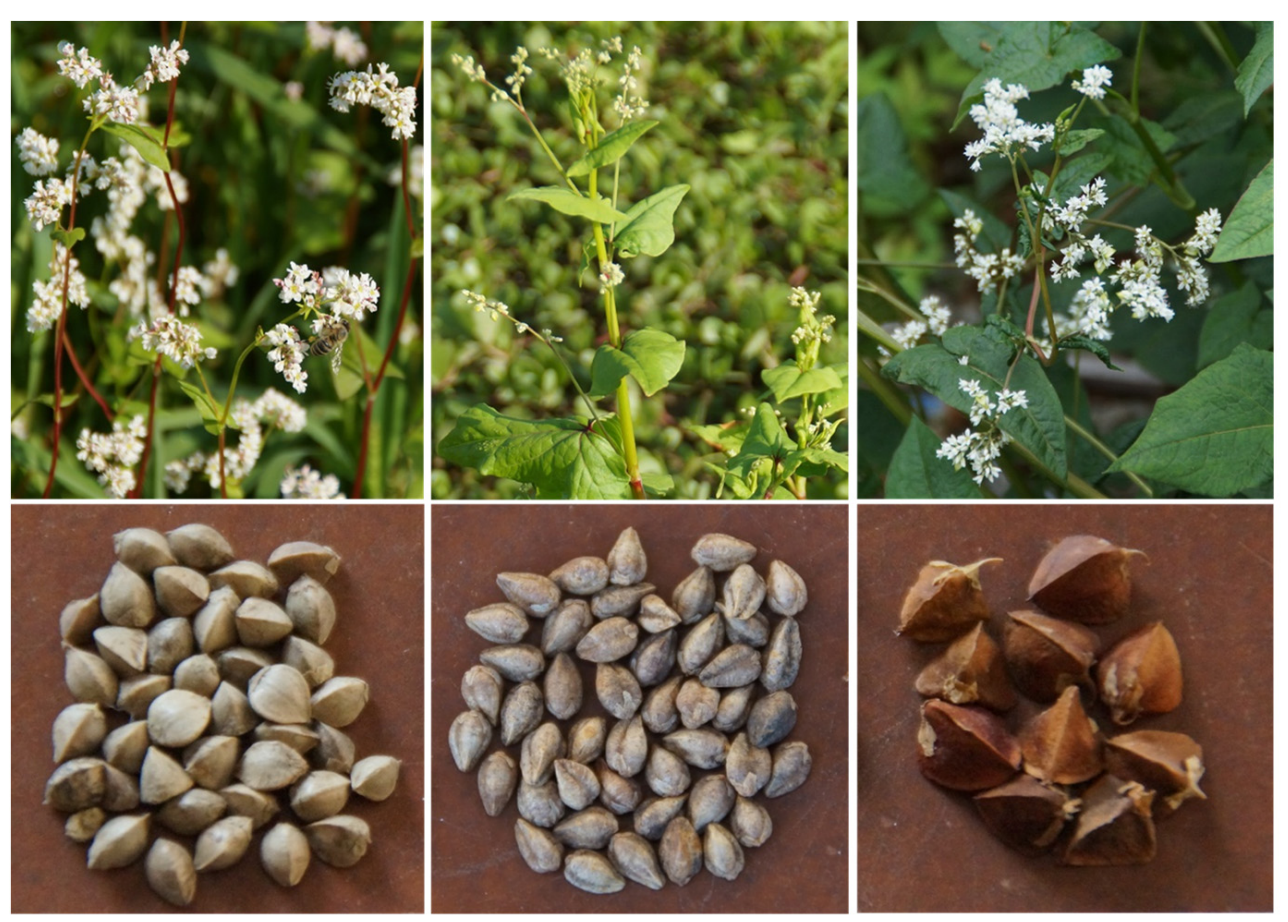
From left to right: plants and grain of common buckwheat, Tartary buckwheat and cymosum buckwheat. Height of plants is normally up to 1.2 m, and grain size is 4 to 6 mm.

Rutin is a flavonoid plant metabolite. Tartary buckwheat is one of the most important nutritional sources of rutin in grain crops [1]. Both common buckwheat (*Fagopyrum esculentum* Moench) and Tartary buckwheat (*F. tataricum* (L.) Gilib.) are used in human nutrition. Among their wild relatives, the wild species *Fagopyrum cymosum* (Trevir.) Meisn. is used in traditional Chinese human and veterinary medicines (Figure 1) [2,3]. Besides appearing in the western parts of China, *Fagopyrum cymosum* is native to India, Nepal, Bhutan, Vietnam and Burma. The area of origin of cultivated buckwheat is the eastern Himalaya region [3,4,5,6]. Tartary buckwheat was included in the interspecific crosses, and several new hybrid species were obtained by crossing them. The best known is *Fagopyrum giganteum* Krotov., from the Ustymivska Experimental Station in Ukraine [7,8]. Among the other important wild buckwheat species is *Fagopyrum homotropicum* Ohnishi, which has been a genetic source in the buckwheat breeding process for obtaining self-pollination in cultivated buckwheat species [9].

**Figure 2:**
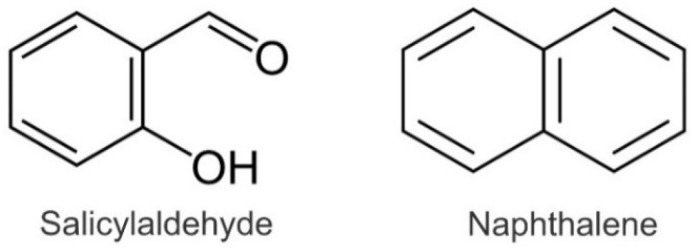
Molecules of salicylaldehyde and naphthalene.

**Figure 3:**
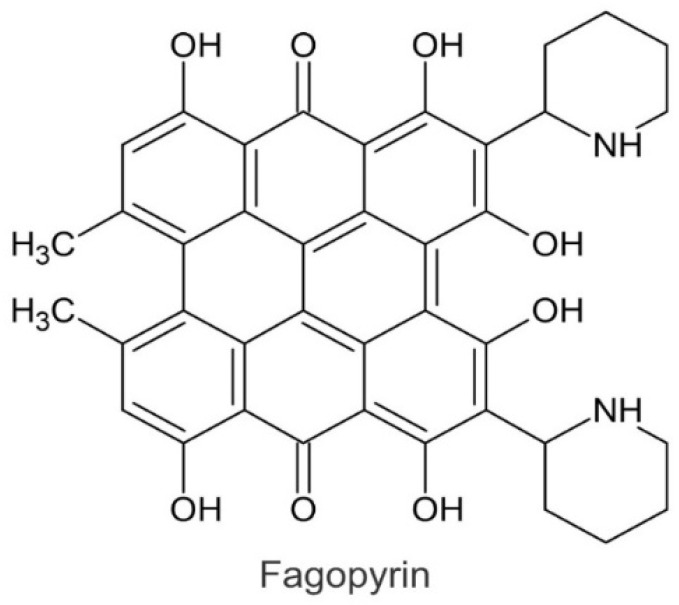
Molecule of fagopyrin.

The strong aroma of Tartary buckwheat grain is characteristic and differs from that of common buckwheat. In the investigations of its phytochemical background, it was established that the most important difference of the Tartary buckwheat aroma in comparison to that of common buckwheat is the presence of naphthalene and the absence of salicylaldehyde in Tartary buckwheat (Figure 2) [10,11]. Common buckwheat, Tartary buckwheat and cymosum buckwheat grain contain the phototoxic metabolite fagopyrin (Figure 3) [9]. Tartary buckwheat is known as an excellent source of high-quality proteins, starch, non-starch polysaccharides, flavonoids and other phenolic substances [12,13,14,15,16,17].

**Figure 4:**
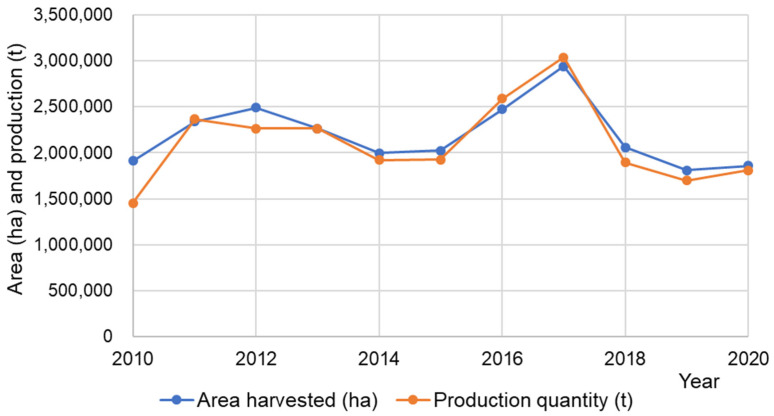
Area harvested (ha) and buckwheat (*Fagopyrum* spp.) production quantity (t) in the world in the last ten years [18].

Buckwheat is grown in Asia, Europe, America and Africa. From the official statistical data (Table 1, Figure 4) [18], it is not possible to distinguish if the data are on common buckwheat or Tartary buckwheat. Authors have observed that, in many countries, it is cultivated common buckwheat with some admixture of Tartary buckwheat. In Bosnia and Herzegovina, the present authors observed the cultivation of a mixture of both buckwheat species in the same field. It is harvested as a mixture, and the seed mixture is then sown again in order to perpetuate the growing of a mixed crop.

The data reported by the authorities of the respective countries are included in Table 1. The authors of this paper have observed the growing of buckwheat as well in Australia, Austria, Italy, Luxemburg, Serbia and Sweden, but, obviously, the FAO has not received data on buckwheat-growing in the mentioned countries. Some buckwheat-growing is possible as well in Afghanistan, Pakistan and the Democratic People’s Republic of Korea, but there are no reliable recent official data available.

Only 19 varieties of Tartary buckwheat are registered with the Community Plant Variety Office (CPVO). Of these, twelve varieties belong to Japan, three to Germany, two to Slovenia and one each to Belarus and Ukraine. There are three varieties in the registration process, of which one is Japanese, one is German and one is Korean [19].

Biotechnological methods involving genetic engineering and newer methods of genome editing (CRISPR, TALEN or ZNF) can be a valuable aid to classical breeding, allowing accurate work with the individual genes responsible for a specific trait. The use of methods with molecular markers, genomics, transcriptomics and proteomics also allows reliable genotype and phenotype studies for the aid in both the breeding of buckwheat and in the identification and production of metabolites important for preserving human health. These newer breeding methods, such as genetic engineering and precise genome editing, which have not yet been widely used in application, except in the case of the in vitro transformation of common buckwheat with an agronomically important Na^+^/H^+^ antiporter gene *AtNHX1*, conferring salt tolerance [20], offer the possibility of obtaining varieties with improved agronomic traits faster and more efficiently.

These approaches have great potential in buckwheat breeding. However, rapid advancements in buckwheat transformation and genome editing provide the opportunity for the development of high-quality, sustainable nutraceutical products through these technologies in the near future. The CRISPR-edited buckwheat products will be expected to have reduced antinutritional factors and enhanced protein, essential amino acids and bioactive compounds compared to the existing commercial cultivars [21]. In several countries in Europe, Tartary buckwheat and common buckwheat are grown as ecological crops. According to the regulations for ecological crops, it is not permissible to use varieties obtained by genetic engineering on such fields or even close to ecological crops. This is the limitation for the use of genetic engineering in the breeding of buckwheat [22].

The aim of this review is to highlight the possible molecular interactions among the primary and secondary metabolites of buckwheat plants in order to optimize the utilization value of products based on common and Tartary buckwheat.

## 2. Flavonoids in Tartary Buckwheat Grain and Herb

Tartary buckwheat grain contains up to 2.4% rutin in the grain dry mass, while the rutin concentration in common buckwheat is about 0.1% and in cymosum buckwheat 1.1% [23]. It was reported that Tartary buckwheat samples from Nepal have a high concentration of rutin in the grain (13.3 g/kg) [24].

**Figure 5:**
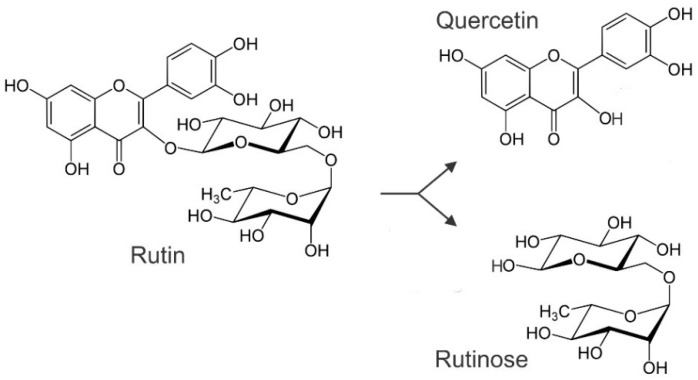
Transformation of flavonoid rutin to aglycone quercetin and rutinose.

Besides the grain, the other aboveground parts of Tartary buckwheat contain rutin as well. The herb of Tartary buckwheat contains up to 4.4% rutin, common buckwheat up to 3.8% rutin and cymosum buckwheat about 4.1% [23]. It was reported that Tartary buckwheat grain contains much more rutin than common buckwheat grain, but it is the same order of magnitude of rutin in Tartary and common buckwheat herb. Tartary buckwheat is, in Asian countries, known as bitter buckwheat as rutin could be easily converted to quercetin, a substance with strong bitterness. The chemical transformation of flavonoid glycosides to aglycones and ethyl-rutinoside in buckwheat is induced by endogenous enzymes that are located in diverse buckwheat tissues (Figure 5). By grain milling, the tissues are disrupted and endogenous enzymes make contact with their substrates [25,26]. In common buckwheat, as the most cultivated buckwheat species, the low concentration of rutin is probably a consequence of the selection based on avoiding bitterness. In contrast to common buckwheat, Tartary buckwheat is better adapted to higher altitudes and harsh environmental conditions [27,28,29,30]. The concentration of phenolic substances seems to be regulated independently in different parts of buckwheat plants, so the selection for a lower concentration in one tissue and, at the same time, an unchanged concentration in the other plant part is feasible [31]. The radiation promotes the synthesis of rutin in common and Tartary buckwheat plants, which protects buckwheat plants against damage by UV radiation [27,28,29,30,32]. The synthesis of protective secondary substances is costly in terms of energy. The synthesis is activated mainly if the damage due to UV-B is greater than the metabolic costs of production of protecting the metabolites [27]. The terminal electron transport system (ETS) activity of mitochondria, providing the energy, is possible to be scored as described by Gaberščik et al. and Kreft et al. [27,33]. In wet conditions, rutin is decomposed to a sugar part and aglycone—quercetin—which is a bitter substance. It is a possibility that, via this method, rutin and quercetin are involved in deterring grazing animals, but there are not yet reports on this kind of protection of buckwheat plants. Rutin deters the probing activities of aphids to reach the non-phloem tissues of plants [34]. The genetic layout of plants and environmental conditions can affect the biosynthesis of rutin and other phenolic substances in the Tartary buckwheat grain and herb [35,36,37].

The extraction of rutin from buckwheat samples is more effective with around 70% ethanol than extraction with more concentrated solvent [38]. This is probably due to the sugar part of the rutin molecule. As rutin and other phenolic substances can be bound to different compounds and grain structures, effective extraction can take several hours [39,40,41]. After milling the Tartary buckwheat grain and mixing the milled product with water, the flavonoid quercetin is obtained in the flour–water mixture, a result of rutin degradation by rutinosidase [41,42,43,44,45]. Heating by hot water or steam inactivates the rutin-degrading enzymes in buckwheat flour. On the contrary, after dry heating buckwheat flour at 150 °C by infrared radiation, the rutin-degrading enzymes remain active for 40 min [46]. Lactic acid bacteria can also split flavonoid glycosides into flavonoid aglycones and sugar. Aglycones could be further metabolised. The products, including lactic acid and other organic acids, are able to increase the antifungal activity of buckwheat dough when used in the production of sourdough bread. In this way, Tartary buckwheat sourdough bread may have a prolonged shelf-life [47].

Of the seventy-four genes potentially related to flavanol synthesis, seven transcript factors have been verified to regulate flavonoid synthesis. Furthermore, it is known that the overexpression of a Tartary buckwheat transcript factor enhances the rutin concentration in vivo. The present research results provide a flavonoid metabolism profile of rutin synthesis. This research is helpful for understanding the molecular mechanisms of rutin synthesis in Tartary buckwheat [48,49].

## 3. Synthesis of Rutin during Germination and Malting

Tartary buckwheat malt and sprouts have a great potential for the production of flavonoids and for functional foods that are rich in flavonoids. They contain up to 54.4 g/kg of rutin [24].

Germinated Tartary buckwheat grain is, according to Bhinder et al. [50], a suitable material for producing functional gluten-free muffins. By the germination process, rutin is, in 72 h, enriched from 1.8 g/100 g db (dry basis) in the grain to 2.1 g/100 g db in the sprouted product, and the quercetin concentration is increased from 0.329 g/100 g to 0.385 g/100 g. During common buckwheat malting (for 144 h) among the studied phenolic compounds, rutin possessed the highest concentration. From 65.17 micro g/g in the grain, it first decreased to 31.83 micro g/g and later increased to 53 micro g/g at the end of malting. The quercetin concentration rose from an initial 3.34 to 6.82 micro g/g [51].

Common buckwheat cold dehusking (without soaking in hot water or steam) was applied for dehusking the seeds. By such a method, the obtained groats maintained the germination ability. Among the phenolic compounds, the most abundant was rutin. It was in the grain with a husk of about 0.1 mg/g db, and, after 96 h of germination following dehusking, 0.9 mg/g db. However, in the sprouts, the most abundant flavonoid was orientin, with a concentration of 2.2 mg/g db after 96 h of germination [52]. Tartary buckwheat sprouts have, in comparison to common buckwheat sprouts, an even better potential as functional food with an excellent composition, including the concentration of total flavonoids. Tartary buckwheat sprouts with plasma-activated water have the flavonoid content of Tartary buckwheat sprouts up to 15.81 mg/g of dry weight after 6 days of germination. This was three times higher in comparison to the flavonoid concentration of the non-germinated grain [53,54].

According to Molinari et al. [55], Tartary buckwheat grain contains a considerable amount of rutin, quercetin, orientin and vitexin. The rutin, quercetin and total flavonoid contents in the raw Tartary buckwheat groats (whole Tartary buckwheat flour) were 2.2, 1.9 and 15.3 mg/g db, respectively. The rutin, quercetin and total flavonoid contents in the Tartary buckwheat germinated for 88 h (whole Tartary buckwheat malt) were 3.7, 4.1 and 16.3 mg/g, respectively. The content of the total flavonoids concentration was higher than the sum of the rutin, quercetin, orientin and vitexin content, which indicated the possibility that there were other flavonoids present in the grain, or the methods for the determination of total flavonoids are somewhat biased.

Tartary buckwheat grain malt could be used to prepare cookies and drinks. The buckwheat malt is rich in orientin, vitexin, rutin and quercetin. The flavonoid concentration in cookies made with Tartary buckwheat material is lower than expected regarding the level in the starting material. The concentration of flavonoids in Tartary buckwheat malt has a higher concentration of rutin in comparison to the grain, soaked or germinated Tartary buckwheat. In the unprocessed Tartary buckwheat, there is 2.2 mg/g db of rutin, and, in whole Tartary buckwheat malt, 3.7 mg/g db of rutin [55]. Plasma-activated water treatment impacts the gradual upward trend in the concentration of flavonoids in the Tartary buckwheat sprouts [54].

## 4. Bioactivity of Flavonoids

Tartary buckwheat metabolites rutin and quercetin are involved in the lipid metabolism [56]. Rutin and quercetin have many pharmacological effects, not just in the blood vessels, muscles and the gastrointestinal system but also in the brain. Namely, blood quercetin is able to cross the blood–brain barrier, and it is accumulated in the brain tissue [57,58]. Quercetin and other phenolics were isolated from the stool samples of people who ingested food rich in phenolic substances. The presence of phenolic substances in the colon can reduce the virus loads in the stools [57]. Rutin has poor solubility, and the absorption is limited in its oral application. A soluble rutin/CH_3_CH_2_OH solvate would be a prospective rutin form for oral preparation according to its better solubility [59].

Hydrogen bonds can connect interactions between rutin and carbon nanotubes, and the complex stabilizes the rutin molecules. The complex of rutin and carbon nanotubes ensures the reduction in cells’ oxidative stress. Therefore, rutin-coated carbon nanotubes are one of the possibilities for delivering rutin to the cells [60,61]. Rutin-loaded nanoparticles of silver are also able to deliver rutin to the site of activity with antithrombotic function [62].

Rutin shows beneficial effects against various inflammatory diseases, and it has neuroprotective effects against oxidative stress in the rat brain [63,64,65]. The phenolic substances extracted from Tartary buckwheat are reported to have an antiproliferative effect on human breast cancer [66] and lung adenocarcinoma [67]. Tartary buckwheat flavonoids show the tendency of inhibiting mammary fibrosis during pregnancy and lactation [68]. Quercetin and rutin are known to possess the potential of the in vitro inhibition of the SARS-CoV-2 main protease [69,70,71]. It is suggested that quercetin could be used with vitamin C for the prevention or treatment of COVID-19 patients in addition to pharmacological agents [72]. Rutin, quercetin and other plant bioactive substances could be retrieved as well from the waste material of the food industry and that of other industries and used for new production and novel purposes [73,74].

## 5. Interaction of Rutin and Its Degradation Products with Proteins

Recently, much attention has been paid to plant proteins in regard to sustainable development and the nutritional importance of plant proteins as they have low carbon footprints. Buckwheat is a low-input plant, adapted to environments that are not suitable for more demanding crops. Buckwheat protein is one of the alternative sources of high-quality plant-based proteins [75]. However, the need to improve the buckwheat protein digestibility is suggested. Improving the buckwheat protein nutritional quality includes the deactivation of allergenic epitopes [76]. Cereal proteins are known to have a reduced digestibility in the presence of phenolic substances [77]. In buckwheat, diverse technologies have an impact on the structural properties and digestibility of proteins [78].

Storage proteins represent about 40% of the total grain proteins in buckwheat [79]. The remaining are functional proteins. In the nutritional studies of diploid common buckwheat cultivar ‘Siva dolenjska’ and the tetraploid common buckwheat variety ‘Bednja 4n’, it was established that the studied buckwheat samples had an excellent amino acid composition, including lysine at the levels 5.0 and 5.2 g/16 g N. The biological value results of the studied buckwheat samples, obtained in the experiments with Wistar rats, were higher than in the high-lysine mutants of true cereals. However, the digestibility of buckwheat proteins was lower than that of wheat. The authors connected the low buckwheat protein digestibility with a high content of phenolic substances [80,81].

The interaction between the proteins and phenolic substances of common buckwheat was confirmed in another experiment with laboratory rats. This interaction appeared during the hydrothermal treatment. As a result of the treatment, the digestion of proteins through the small and large intestine was reduced when animals were treated with antibiotics to prevent the activities of microbes in the large intestine. In the animals not treated with antibiotics, the large intestine microorganisms digested the proteins that would otherwise be blocked by phenolic substances due to the hydrothermal treatment of common buckwheat [14].

Common buckwheat milling fractions may have diverse concentrations of proteins and phenolic substances [16]. In the fraction with the highest concentration of proteins (31%), it was 475 ppm rutin and 6% of tannins. In one of the starch-rich fractions (about 90% starch), it was only 19 ppm rutin, 0.1% tannins and 4.4% proteins. The milling of buckwheat grain to milling fractions with diverse concentrations of nutrients could become a novel processing technology for delivering materials for preparing functional healthy foods.

In buckwheat grain, the protein content and total polyphenols content in both free and bound polyphenols gradually decreased from the outer to the inner fractions [82]. This result was obtained by gradual surface abrasion. The protein-rich fraction was, via this method, enriched with a polyphenol concentration of up to 55 mg/g and a protein concentration of up to 36%. After treatment with hot steam at 130 °C and being cooled down, the high temperature of the hydrothermal process may cause the denaturation of proteins, with decreased availability of proteins for enzymatic actions. The decrease in protein digestibility could be connected with the restricted accessibility of proteins to enzymes in the more rigid protein network [83].

**Figure 6:**
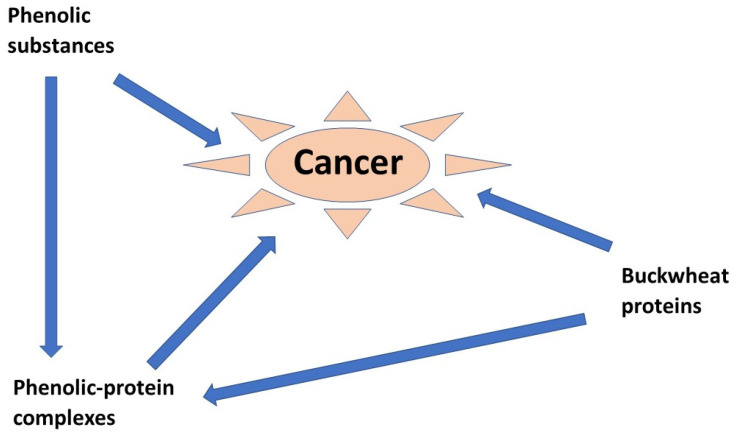
Pathways of alleged suppression of appearance of cancer, according to the references (Refs. [66,67,68,91,92,97]).

Phenolic compounds have low absorption after food intake, so, after ingestion, they remain for some time in the gastrointestinal tract. They can act in an inhibitory manner on enzymes degrading proteins [84]. This inhibition of protein digestion is not advantageous from the point of view of the availability of amino acids for the needs of the body. However, slowly digestible proteins have several desirable effects [85,86]. The quality of buckwheat products and their nutritional functionality depend on the presence of proteins and peptides [87]. Buckwheat proteins ameliorate constipation problems in rats and reduce the hepatic triglyceride concentration [88,89]. Interesting results were obtained in experiments with rats that were fed extracted buckwheat protein. The results showed that buckwheat proteins counteract mammary carcinogenesis [90]. The anticancer activities of rutin, quercetin and other buckwheat metabolites have been studied by in vitro experiments in regard to the inhibition capacity on the growth of cancer cells, or by effects in experimental animals with chemically induced cancers (Figure 6) [66,67,90,91,92]. Promising results were obtained in rats fed a buckwheat protein extract as the source of protein. Compared to the rats fed casein, the buckwheat protein diet reduced the body fat and caused muscle hypertrophy [93,94,95,96].

In buckwheat grain, there are different levels of phenolics, which could react with proteins [98]. Ikeda et al. [99] studied buckwheat plant antinutrients and their impact on the digestibility of buckwheat proteins. Among the trypsin inhibitors are known buckwheat plant defence peptides [100].

Hydrolysed buckwheat proteins contained di-, tri-, and tetrameric peptides. Based on the content of tryptophan, proline, valine, leucine and phenylalanine, the proteins show a strong radical scavenging activity. Buckwheat albumin is very rich in antioxidant amino acids; they demonstrate antioxidant activity as well when the proteins are digested to peptides [101]. Buckwheat products may contain important blood-pressure-lowering peptides, obtained from fermented buckwheat sprouts [95,102,103].

Many other bioactivities of buckwheat proteins are known. Phenolic-protein complexes act as radical sinks [104]. They are involved in angiotensin-I-converting enzyme inhibitors [105] and in diverse anti-tumour activities (Figure 6) [97] and antimicrobial potential [106]. Buckwheat protein suppresses the plasma cholesterol concentration in hamsters and prevents the formation of gallstones more intensively than soybean protein [107,108,109].

Another important function of proteins is that they are able to include selenium amino acids as the selenium storage. However, proteins with selenium amino acids in place of non-selenium variants may have different activities or functions [110]. Common and Tartary buckwheat treated with selenium could be a rich source of this essential element [29,32].

## 6. Interaction of Flavonoids with Starch

The digestibility of gelatinized starch depends on the starch structure [111]. The Tartary buckwheat starch digestibility is reduced by rutin and quercetin as they alter the starch structure and inhibit the activity of digestive enzymes. Quercetin has better enzyme inhibition than rutin [112]. The interactions between Tartary buckwheat starch and quercetin probably result from hydrogen bonding with a weak hydrophobic force [113]. In abrasive milling fractions, the starch digestibility and bioaccessibility of the buckwheat protein were increased with hydrothermal treatment [82]. After the hydrothermal treatment, the structure disruption of the buckwheat protein flour enhanced the digestibility of the starch and biological accessibility of phenolic substances with the time of impact. This route of the dry surface abrasion process in combination with hydrothermal treatment demonstrates promising ways to obtain attractive buckwheat products. It was established that the starch digestibility is higher after the hydrothermal treatment of buckwheat than in untreated flour. Generally, when the flour is treated by steam at an elevated pressure and is suddenly exposed to atmospheric pressure, the steam in the flour particles quickly expands and the steam disrupts the structures, producing holes in the material [77]. Some nutrients, such as starch and protein, can bind to phenols in complexes, which can slow the digestion [12,13,114,115,116].

The rutin and quercetin in Tartary buckwheat grain have an impact upon the physicochemical properties of the starch after cooking. The aging enthalpy of retrograde starch is lowered, and the viscosity of the Tartary buckwheat starch and paste is increased. Starch-phenolic binding is stronger than that of the complex of starch and iodine. Starch is gelatinised and retrograde, and the morphology is affected by quercetin and rutin [115].

The slow digestion properties of starch were studied by Luo et al. [116] following the ethanol extract of Tartary buckwheat. The slow digestibility of this starch appeared to be due to the impact of the phenolic substances on the starch. In their in vivo experiments, mice demonstrated reduced postprandial glycaemic responses. These data of Luo et al. [116] for Tartary buckwheat grain and glycaemic responses were similar to those obtained earlier in common buckwheat [114].

As mentioned, common buckwheat milling fractions may have diverse concentrations of constituents, including starch. In starch-rich fractions, it could be over 90% of the starch [16]. Such products are of lesser importance from the nutritional point of view. However, small buckwheat starch granules are hydrophobic, and they are, in contrast to large wheat or other true cereal starch granules, rarely damaged during milling. Buckwheat starch granules are hydrophobic, and they are potentially a suitable material for producing fat replacers [117]. In the traditional processing of Japanese buckwheat “soba” noodles, the milling fraction with a high starch content is known as “sarashina” buckwheat flour, and it is used to prevent the buckwheat dough layers from sticking after rolling out, or to prepare “sarashina-soba”, white buckwheat noodles [118]. The amount of resistant starch is affected by the composition of the starch in terms of its high amylose content and depending on ecological and genetic factors [117].

In Tartary buckwheat, the quercetin complexation with starch molecules has an impact on the in vitro digestibility of the starch and the appearance of resistant starch, thus altering the physicochemical properties of the Tartary buckwheat starch [119]. The effects of this quercetin–polyphenol complexation indicate that food products based on Tartary buckwheat will show lower digestibility. Indeed, the quercetin in Tartary buckwheat can reduce body weight, serum triacylglycerols and low-density lipoprotein. In rats, a diet with 0.1% quercetin was shown to significantly lower the low-density lipoprotein concentrations in the serum, with no such effects on the high-density lipoprotein concentrations. Tartary buckwheat has also been shown to prevent increases in body weight and fat deposition during high fat intake in rats, although, on the other hand, this was reported to protect against hepatic stenosis [56]. A buckwheat diet can also reduce insulin and ameliorate glucose intolerance in humans [114].

Rat experiments with common buckwheat have further suggested the complexity of the impact regarding the gut microbiota [12,13,14]. Indeed, Peng et al. [56] suggested that the link between weight gain and the gut microbiota is very complex, with the need for further studies to be conducted in the future.

Interestingly, it has been shown that rutin-enriched Tartary buckwheat flour extracts provide better flavonoid oral absorption, with the phenolic substances in the blood detectable for longer than with standard rutin, and even longer than for a native Tartary buckwheat grain flour extract [76]. Rutin is, for the most part, bound to other grain substances and structures. Indeed, the extraction of rutin from untreated Tartary buckwheat grain flour showed 0.57 g rutin/100 g flour, while autoclaving resulted in 3.03 g/100 g flour, boiling resulted in 2.97 g rutin/100 g flour and steaming resulted in 2.50 g/100 g flour [76,120]. Dzah et al. [120] also studied solid–liquid extraction conditions for Tartary buckwheat, where they indicated that the extraction of the phenolic compounds from Tartary buckwheat flour can be performed at <65 °C [121].

During the process of hydrothermal preparation, which is a traditional way of preparing buckwheat groats, phenolic substances migrate with hot water from the grain pericarp into the inner parts. Therefore, in dehusked groats, there are more phenolic substances than originally in the inner part of the grain or in the groats that are dehusked without water and temperature treatment [10,11]. In processing buckwheat grain for different food products, various interactions are possible among the constituents, especially during hydrothermal treatments. Tartary buckwheat noodles, prepared by using extrusion technology, showed lower estimated glycaemic index values and reduced the level of total cholesterol, triacylglycerols and low-density lipoprotein cholesterol but increased the concentration of high-density lipoprotein cholesterol [122].

## 7. Conclusions

In this paper, new insights on the importance of buckwheat constituents for nutritional value, functional food products and the health of consumers were presented. Buckwheat plant metabolites are in the plant parts included in the matrix of cells, cell walls and botanical structures. By milling, thermal, hydrothermal and other treatments, the molecules leave the original matrix and are disposed to enter into the new aggregates and other structures, with different solubility, bioavailability and effects on the human body.

The dimension of time elapsed is essential for the exit of metabolites from the original site and to enter into the new molecular states or molecular aggregates. Weak molecular bonds are essential in this process. Rutin, quercetin and other buckwheat metabolites have a weak solubility in water or other solvents, but the presence of different molecules in the solution may enhance or otherwise change their solubility.

Tartary buckwheat is an excellent source of high-quality proteins, starch, non-starch polysaccharides, flavonoids and other phenolic substances. Among the important flavonoids is rutin. It is present in wet conditions and, at a moderate temperature, degrades to quercetin. At high temperatures, this process is stopped as the rutin-degrading enzymes lose their activity.

The synthesis of rutin and other protective secondary metabolites is costly for plants in terms of energy. The synthesis is activated mainly in the presence of UV-B radiation or other threats to the plants.

Rutin shows beneficial effects against various inflammatory diseases, and it has neuroprotective effects against oxidative stress in the rat brain. The phenolic substances extracted from Tartary buckwheat are reported to have an antiproliferative effect on human breast cancer and have protecting effects against the appearance of other types of cancers.

Rutin is able to build complexes with other molecules. The interaction between quercetin and Tartary buckwheat starch is probably by hydrogen bonding with a weak hydrophobic force. The slow digestibility of buckwheat starch appeared to be due to the impact of phenolic substances on the starch. The Tartary buckwheat starch digestibility is reduced by rutin and quercetin as they alter the starch structure and inhibit the activity of starch-degrading enzymes. Quercetin has better enzyme inhibition than rutin. In Tartary buckwheat, the quercetin complexation with starch molecules has an impact on the in vitro digestibility of the starch and the appearance of resistant starch.

Buckwheat protein is able to suppress the plasma cholesterol concentration and formation of gallstones more intensively than soybean protein. High protein buckwheat flour products suppress hypercholesterolemia and gallstone formation; this is connected with the low buckwheat protein digestibility due to the complexation of proteins with phenolic substances.

Based on the content of tryptophan, proline, valine, leucine and phenylalanine, buckwheat proteins and peptides have a strong radical scavenging activity. Buckwheat albumins are especially very rich in antioxidant amino acids, and they possess antioxidant activity as well when proteins are digested to peptides.

Hydrogen bonds can connect the interaction between rutin and carbon nanotubes; the complex stabilizes the rutin molecules. The complex of rutin and carbon nanotubes ensures a reduction in cells’ oxidative stress. Therefore, rutin-coated carbon nanotubes are one of the possibilities for delivering rutin to the cells.

Common and Tartary buckwheat food products have a lower glycaemic index and reduce the level of total cholesterol, triacylglycerols and low-density lipoprotein cholesterol but increase the concentration of high-density lipoprotein cholesterol. This is a good starting point for the development of functional foods and pharmaceutical products based on common and Tartary buckwheat.

In this review, some of the possible molecular interactions among the primary and secondary metabolites of buckwheat plants were highlighted in order to optimize the utilisation value of products based on common and Tartary buckwheat. Further research on the molecular levels of the metabolites and on their solubility, digestibility, bioavailability and impact on human health are needed.

## Figures and Tables

**Table 1:** Crops of buckwheat (*Fagopyrum* spp.) by countries and in the world [18].

Country	Year of Data	Area Harvested (ha)	Yield (t/ha)	Production Quantity (t)
Belarus	2020	27,354	1.03	28,300
Bhutan	2020	2004	1.35	2701
Bosnia and Herzegovina	2020	833	1.56	1301
Brazil	2020	46,416	1.40	65,117
Canada	2020	9800	0.91	8900
China, mainland	2020	624,780	0.81	503,988
Croatia	2017	695	0.90	624
Czech Republic	2017	887	2.55	2262
Estonia	2017	5278	0.64	3385
France	2017	74,883	3.52	263,485
Georgia	2020	106	1.11	118
Hungary	2017	969	0.94	909
Japan	2020	66,600	0.67	44,800
Kazakhstan	2020	55,076	0.73	40,094
Kyrgyzstan	2020	10	1.70	17
Latvia	2017	18,300	0.93	17,100
Lithuania	2017	48,499	1.10	53,221
Nepal	2020	10,369	1.13	11,724
Poland	2027	78,027	1.45	113,113
Korea	2020	1600	0.97	1549
Republic of Moldova	2020	5	0.80	4
Russian	2020	821,366	1.09	892,160
Slovakia	2017	429	0.86	367
Slovenia	2017	3647	0.80	2909
South Africa	2020	579	0.40	234
Ukraine	2020	84,100	1.16	97,640
United Republic of Tanzania	2020	24,295	1.06	25,772
USA	2020	81,620	1.06	86,397
World		2,088,527	1.16	2,268,191

## Data Availability

Not applicable.
